# Supplementary figures and images for: MixInYeast: A Multicenter Study on Mixed Yeast Infections
Source: J Fungi (Basel). 2020 Dec 29;7(1):13. doi: 10.3390/jof7010013 (PMC7823447; doi:10.3390/jof7010013)

## Supplementary material

Figure S1. Flow chart of the MY isolates analyzed

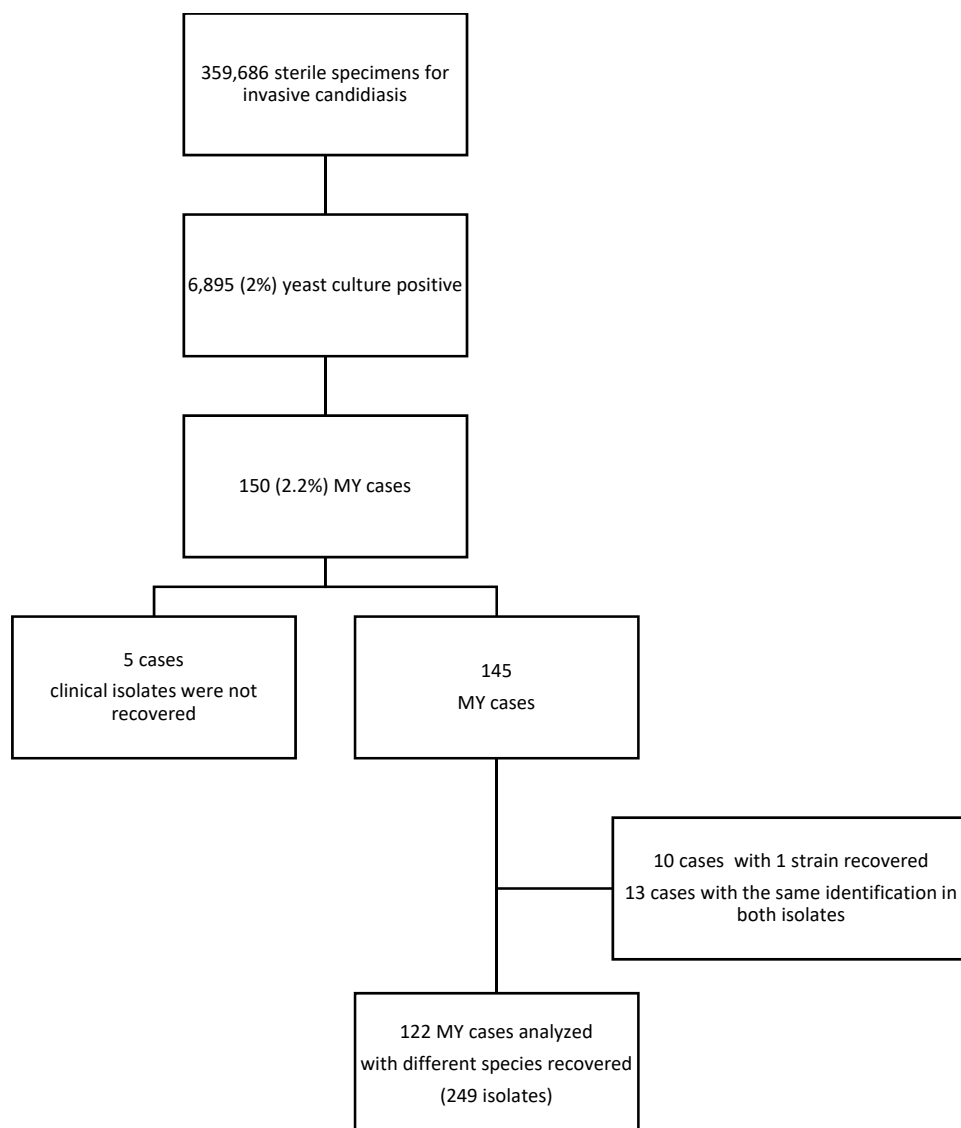

Supplement: Supplementary file 1 [file jof-07-00013-s001.pdf]
